# An assessment of autistic and parkinsonian movement profiles to inform selective classification algorithms

**DOI:** 10.1186/s11689-025-09668-8

**Published:** 2026-01-10

**Authors:** Lydia J. Hickman, Dagmar S. Fraser, Joseph M. Galea, Francesca Happé, Jennifer L. Cook

**Affiliations:** 1 MRC Cognition and Brain Sciences Unit, University of Cambridge, 15 Chaucer Road, Cambridge, CB2 7EF UK; 2 Centre for Human Brain Health, School of Psychology, University of Birmingham, Birmingham, B15 2TT UK; 3 Social, Genetic and Developmental Psychiatry Centre, Institute of Psychiatry, Psychology and Neuroscience, King’s College London, London, SE5 8AF UK

**Keywords:** Autism, Parkinson’s disease, Movement, Classification

## Abstract

**Background:**

Movement differences in autism have attracted growing attention in recent years. Anecdotally, autistic movement has been likened to that of Parkinson’s Disease (PD). Given that PD assessments are primarily movement-based, it is important to ensure that autistic individuals are not scoring highly on PD diagnostic criteria due to autism-related movement differences. Quantifying overlap in movement profiles and identifying distinguishing features is essential, particularly given increased PD diagnosis rates in the autistic population.

**Methods:**

We conducted the first direct comparison study of autistic and parkinsonian movement. Autistic individuals (*N* = 31), individuals with PD (*N* = 32) and control participants (*N* = 31) completed a Shapes Tracing Task and a Reaction Time Task. Kinematic features were compared between groups and classification algorithms were run to distinguish between groups.

**Results:**

Groups were distinguishable based on kinematic features. The autistic group differed from both PD and control groups in speed modulation and sub-movements, and from the PD group in reaction time. Classification algorithms for clinical (autism and PD) versus non-clinical groups, and for autism versus PD, were most accurate when combining kinematic and questionnaire data. There were no kinematic similarities between autism and PD that were also distinct from controls.

**Conclusions:**

Whilst kinematic features did not appear similar between autism and PD, they were informative for group classification. This proof-of-concept study highlights that movement-based metrics may aid in identifying whether someone belongs to a clinical group, and which one – suggesting potential for refining diagnostic approaches for both autism and PD.

**Supplementary Information:**

The online version contains supplementary material available at 10.1186/s11689-025-09668-8.

## Background

Whilst the majority of autism [1] research has focused on social communication, and restricted and repetitive interests [2], the field of motor function has garnered attention in recent years. A growing body of evidence has highlighted differences between autistic and non-autistic individuals in motor planning [3, 4], execution [4, 5] and coordination [6]; indeed, meta-analyses have evidenced differences across a variety of motor tasks [7, 8]. Such differences are apparent from a young age, with both delayed motor development and motor function atypicalities reported in autistic children [9]. As such, calls have been made to include movement differences as “specifiers” to autism diagnoses (i.e., condition-relevant but non-selective symptoms used to further clarify a diagnosis; [10]).

Autistic movements have been informally likened to those observed in Parkinson’s Disease (PD), particularly in core domains such as bradykinesia (slowed movement), gait differences and postural instability [4, 11, 12]. Apparent similarities extend beyond the motor domain, including emotion production [13–15], theory of mind [16, 17] and cognitive rigidity [18–20]. These overlapping traits prompted several reviews to highlight similarities between autism and PD at genetic and neurobiological levels [21–24]. In particular, dopamine dysfunction has been implicated in both conditions [25, 26], a finding of clear relevance when considering the origins of motor similarities. This growing body of work has led to calls for direct comparisons of autism and PD in order to better understand the nature of their relationship [24]. 

Similarities between autism and PD have the potential to obscure diagnostic processes. Elevated PD diagnosis rates are observed in the autistic population [22, 27–30]. However, it is also the case that autistic individuals score highly on the gold standard PD diagnostic assessment: the Movement Disorders Society Unified Parkinson’s Disease Rating Scale (UPDRS; 27, 31). It is important to question whether this is simply due to the presence of autism-related movement differences which *appear* parkinsonian in such assessments as opposed to genuine co-occurrence of conditions [24]. To answer this question, a first step is to quantify the extent to which autistic and parkinsonian movement overlaps. This is particularly relevant in the context of new classification algorithms for people with and without PD, which use kinematic features extracted from fine motor function [32–40]. If autistic and parkinsonian movement is similar at a fine-grained level, autistic individuals are at risk of being categorised into the PD group in classification algorithms. Whilst studies have demonstrated the utility of movement kinematics for classifying autistic and non-autistic groups [41–43], this has not been implemented alongside the classification of other movement disorders such as PD. 

Whilst no direct comparisons of autistic and parkinsonian movement have been conducted, reviewing the respective literature indicates potential fine motor function similarities including jerky movements (i.e., rapidly changing acceleration and/or deceleration; 44–47]) and an increased *number* of alternations between acceleration and deceleration (or “sub-movements”; [46, 48–52]). Nevertheless, there is also evidence of movement differences: whilst autistic individuals have been found to move with increased velocity and acceleration [44, 47, 48], and with reaction times that are comparable to the general population [53], individuals with PD exhibit slower movements, lower acceleration, and longer reaction times [46, 49, 50, 54–60]. A further kinematic feature of interest is speed modulation: the adaptation of movement speed based on the curvature of a trajectory. Individuals generally speed up along straight parts of a shape and slow down around corners, leading to a negative relationship between speed and the curvature of a trajectory [61]. Interestingly, speed modulation has been found to be higher in autistic populations (a sharper slowing down of speed around tight corners; [62, 63]) and lower in low dopamine conditions (a more gradual adaption of speed; [64]) such as PD. Overall, separate research studies indicate the likelihood of both similarities and differences between autistic and parkinsonian movement.

At present, comparisons of autistic and parkinsonian movement requires the utilisation of findings from separate studies with different experimental designs, each varying with respect to participant demographics. This is problematic because kinematic features are known to be influenced by a range of factors. For example, slower movement is seen with demographic factors such as increasing age [65] and lower IQ [66], and clinical traits such as depression and anxiety [67, 68] which are known to be elevated in autism [69] and PD [70, 71]. Indeed, the movements of those with melancholic depression have been quantitatively likened to those of PD [67]. A primary objective of the current study was to directly compare autistic and parkinsonian movement in a single controlled experiment, either matching or, where matching was not possible, controlling for these variables.

Beyond comparing autistic and parkinsonian movement, a further aim of the study was to assess the utility of kinematic features in classifying autism, PD and control groups, above and beyond common questionnaire measures. This is potentially useful for two reasons. Firstly, fine-grained kinematic features may be identified which can differentiate between autism and PD, with the longer-term potential of using such measures alongside standard assessments (e.g., the UPDRS) to refine the characterisation of movement disorders. Secondly, existing movement-based algorithms, as mentioned previously, typically focus on one clinical condition and do not incorporate other movement conditions (though note Duque and colleagues’ differential diagnosis method for PD versus essential tremor; [72]). Therefore, we addressed this issue by using movement features to first identify whether someone might belong to a clinical group (here, autism or PD) or non-clinical group and, subsequently, *which* clinical group they belong to.

In the current study, we compared the kinematic features of three groups of older adults: autistic individuals, individuals with PD and control participants. To index kinematics, participants traced set movement trajectories – shapes that have a highly predictable relationship between speed and curvature when traced by members of the general population [61] – on a touchscreen device. Kinematic features were extracted from participants’ recorded movements and a Reaction Time Task was used to index reaction time. Here, we present the first comparison of kinematic features between these groups on a task of restricted movement, and assess the utility of such kinematic features in predicting group membership.

## Methods

### Participants

We recruited three groups of participants: autistic individuals (ASD; *N* = 31), individuals with PD (PD; *N* = 32) and control participants (CTRL; *N* = 31). Participants in the ASD and PD groups were required to provide evidence of a formal clinical diagnosis (e.g., a letter from a clinician) or were recruited from existing participant databases in which such evidence had already been verified. Participants who reported a diagnosis of co-occurring movement or developmental disorders were excluded. Recruitment channels included Parkinson’s UK, Autistica, the University of Birmingham Psychology Autism Research Database, the University of Birmingham Older Adults Database, and social media. The PD group was recruited first as an opportunity sample. Older autistic adults were then recruited with the aim of matching the PD group, and the control group was recruited to match both clinical groups as closely as possible. Gender was readily matched, whereas age matching was limited by the older age of PD participants and the relative scarcity of older adults with autism diagnoses [73]. We aimed to match depression and anxiety symptoms across groups, rather than excluding these conditions (due to their strong co-occurrence with autism and PD). Eight participants in the ASD group and six participants in the PD group were taking psychoactive medication at the time of testing. All participants gave fully informed consent in accordance with the Declaration of Helsinki and received remuneration of £10 per hour. The experimental procedure was approved by the local Research Ethics Committee (ERN_18-1800B and ERN_16-0281AP5).

### Procedure

Participants first completed an online screening form followed by a set of questionnaires and the Matrix Reasoning Item Bank (MaRs-IB; [74]). Subsequently, participants completed two testing days at home. On each testing day, participants completed a Reaction Time Task and the Shapes Tracing Task using a stylus and touch-screen device (Samsung Galaxy Tab A7; 10.40-inch touchscreen; 2000 × 1200 pixels). The tasks were programmed in PsychoPy and run on Pavlovia (PsychoJS platform version 2021.1.4). Participants with PD completed these tasks prior to taking their first dose of dopaminergic medication in the morning; this protocol achieved OFF-medication state and is standard practice in the literature [75–77]. Note that whilst performance was also recorded on a separate day ON-medication (orders were counterbalanced across participants), only OFF-medication data is analysed here given the aim to assess naturalistic PD movements. For reference, the PD medications taken by participants are detailed in Appendix 1 (Table A1). To control for practice effects, ASD and CTRL participants completed the tasks on two separate days and *day* was included as a factor in subsequent analyses (see Appendix 6 for analyses which only include data from one day for the ASD and CTRL groups). Testing days were no longer than three days apart for any participant.

#### Online questionnaires

Following demographic questions to check eligibility (i.e., official diagnosis of ASD or PD) and to facilitate group matching (e.g., age, gender), participants completed the Autism Quotient (AQ; [78, 79]) and the Ritvo Autism Asperger Diagnostic Scale (RAADS; [80]) as measures of autistic traits, Sect. 2 of the UPDRS (Part II: Motor Aspects of Experiences of Daily Living; [81]) as a measure of parkinsonian traits, the Patient Health Questionnaire (PHQ; [82, 83]) as a measure of depression, and the Generalised Anxiety Disorder Assessment (GAD; [84]) as a measure of anxiety.

#### Matrix reasoning item bank

Participants completed the MaRs-IB on Gorilla. The task lasted 8 min and in each trial participants had to select the appropriate shape to fill the empty cell of a 3 × 3 matrix. Scores were calculated as the proportion of correct responses within 8 min. The task is a validated measure of non-verbal reasoning ability, and such a task-specific measure has been argued to be most appropriate for group matching autistic and non-autistic samples [85, 86].

#### Reaction time task

The Reaction Time Task included 8 practice trials and 40 experimental trials. Participants were asked to use a stylus held in their dominant hand and hover over the centre of the tablet before each trial. Four target locations were present on the screen: 2.5 cm by 2.5 cm white boxes, spaced 1 cm apart and centred on the tablet. Participants were instructed to press the target (a black X) as soon as it appeared in one of the boxes. Response time was recorded from target appearance to touch.

#### Shapes tracing task

The Shapes Tracing Task was used to index participants’ movement kinematic features. Participants used a stylus and touch-screen device to trace shapes in a counter-clockwise direction for 10 full cycles per trial. Each shape was scaled to its maximum possible size while ensuring that neither dimension exceeded 9 cm. A light grey visible trace marked the stylus movements made by the participant, to facilitate accurate tracing. They were instructed to make these movements “as fluidly as possible”, using a stylus in their dominant hand. Whilst this instruction was in line with previous literature employing the task [44, 61], subsequent guidance advocates the use of both a ‘naturalistic’ movement speed and an ‘increased’ pace [87].

**Fig. 1:**
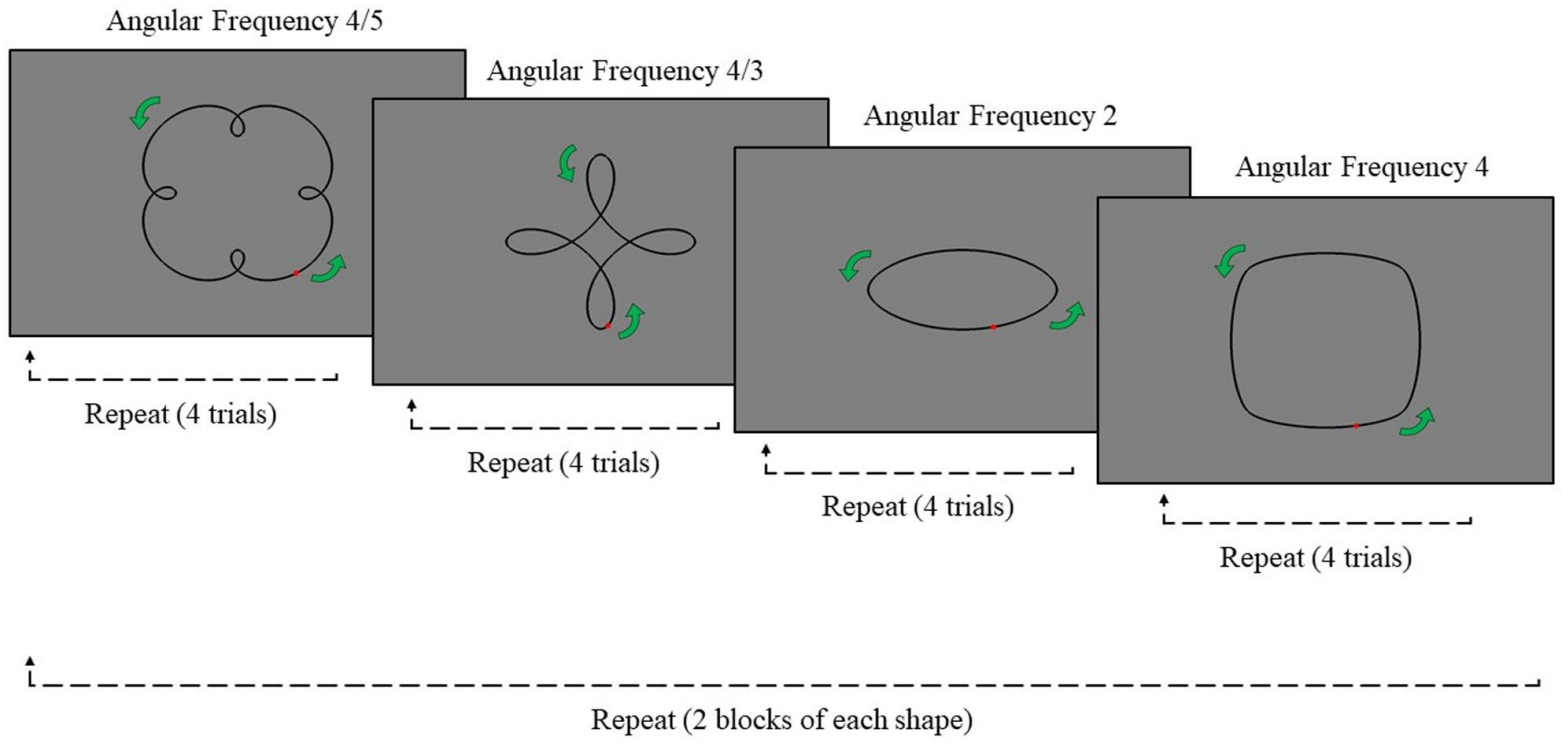
The shapes tracing task trial appearance. Note. The four shapes differed with respect to their angular frequency (AF): clover (AF 4/5), petals (AF 4/3), ellipse (AF 2), and rounded square (AF 4). Participants were instructed to begin tracing the shape at the red dot and trace in the direction of the green arrows. A total of eight blocks were completed, two of each shape. Blocks were presented to participants in a random order

Four shapes were traced during the task to obtain movement across a range of trajectories (Fig. 1). These shapes spanned a range of angular frequencies (i.e., the number of curvature oscillations per two π of angular displacement): 4/5 (“clover”), 4/3 (“petals”), 2 (“ellipse”) and 4 (“rounded square”). These symmetrical shapes represent core components of movement trajectories and as such are the constituent parts of more complex naturalistic movements such as handwriting.

Eight blocks were completed, two for each shape, in a random order. Within each block, seven attempts could be made to complete four successful trials. Thus, a maximum of eight successful trials was possible per shape. Trials were deemed “unsuccessful” if, following a 5-second grace period, participants lifted their stylus from the screen or deviated too far from the shape. This boundary was determined through pilot testing to be sufficiently small to prevent participants from drawing shapes that deviated substantially from the intended trajectory, yet large enough to ensure the task remained achievable for clinical groups. Participants were notified of unsuccessful trials, and the reason was stated. A timeout of 90 s was set for each trial.

### Data pre-processing

#### Reaction time task

Scores for the Reaction Time Task were calculated as the average response time (ms) after the appearance of an ‘X’ across the 40 experimental trials. Outliers, values further than 2 standard deviations away from the mean, were removed (3.7% of data).

#### Shapes tracing task

X and y coordinates of the stylus position over time were used to calculate kinematic features for each trial. Trials with fewer than 2.5 traces of the shape were removed. The first ½ π angular displacement of each trial was discounted before data processing. Samples which did not achieve a minimal speed (200 pixels per second) within the 5-second grace period were removed. The device had a maximum sampling rate of 60 Hz but, given deviations, positional data was resampled using the spline method to achieve a consistent 60 Hz.

Speed, acceleration and jerk were calculated as the first-, second-, and third-order derivatives of the positional non-null data. That is, speed refers to the distance moved (in pixels) over time (in seconds); acceleration refers to the change in speed over time; jerk refers to the change in acceleration over time. These values were obtained using a smooth differential filter at each stage of differentiation [61]. Units are converted from pixels to mm for box plots in Appendix 7.

Sub-movements were calculated as the percentage of time samples (recorded at 60 Hz) in a given trial in which a change in acceleration sign was observed.

**Fig. 2:**
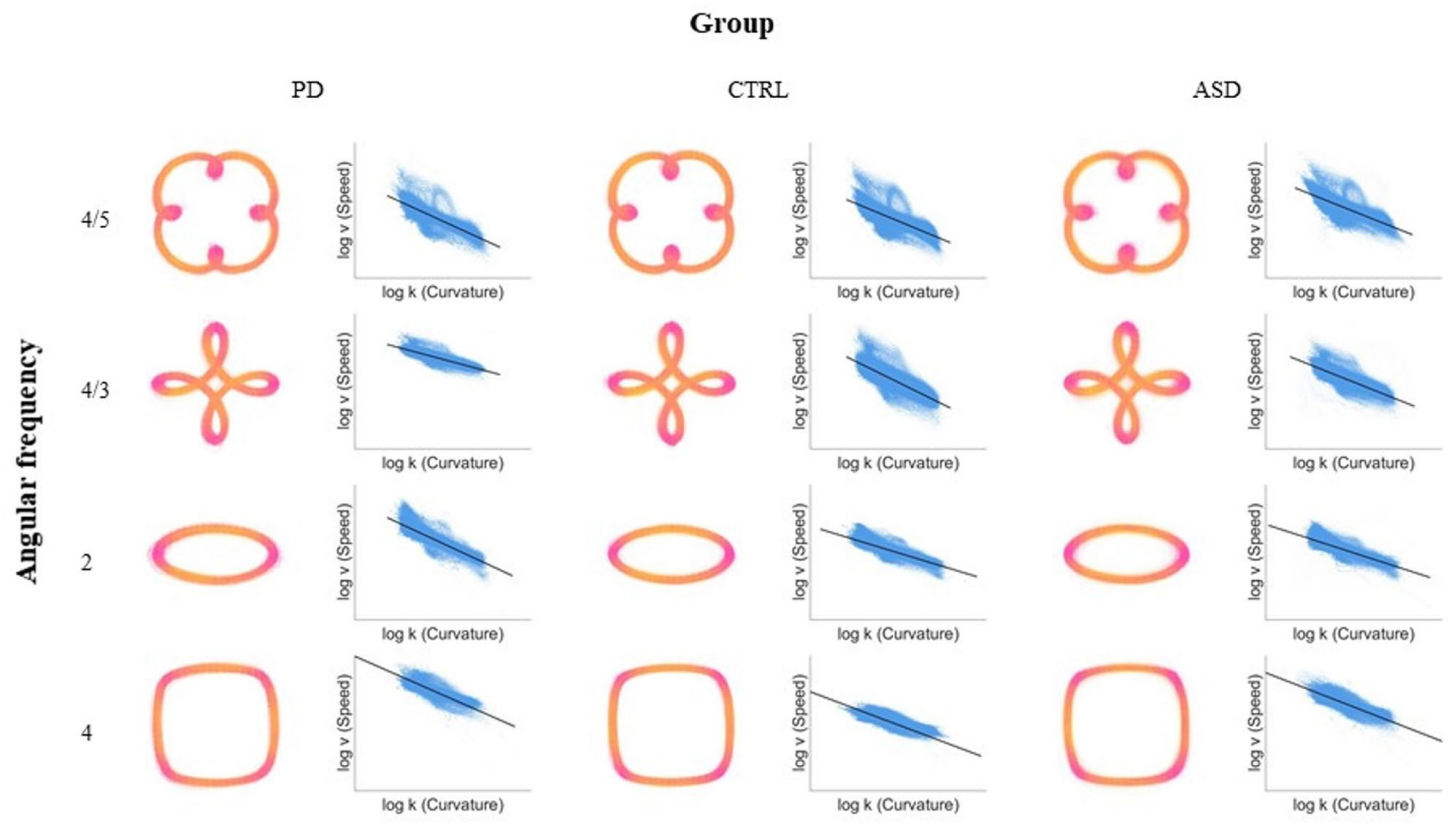
Shapes tracing task raw movement trajectories and speed-curvature plots. Note. For each group and shape, two plots are presented. Raw movement trajectory plots (left) depict the x and y coordinates of the stylus position throughout the trial (for all trials), with the colour scheme representing speed at those positions from dark pink (low speed) to yellow (high speed). Speed-curvature plots (right) depict speed values (log v) plotted against instantaneous curvature values (log k) for all trials, as obtained following the filtering procedure detailed in the Methods section. A black regression line is fitted to the data. As can be seen, individuals typically speed up along straight parts of a shape and slow down around corners, leading to a negative relationship between speed and curvature. Axes are scaled automatically for each panel to optimise visualisation of the speed-curvature relationship; therefore, speed values are not directly comparable across shapes or groups (see Figs. 3 and A1 for group- and shape-level comparisons of speed).

Speed modulation values (also known as speed-curvature gradients; $\:\beta\:$ in Eq. 1) were calculated as the gradient between tangential velocity ($\:v$ in Eq. 1) and the current curvature of the shape being drawn ($\:\kappa\:$ in Eq. 1), converted to an absolute value. This followed the established filtering and regression procedure [61, 62, 87], including a Savitzky-Golay filter to smooth the data and reduce high-frequency noise. A higher speed modulation value indicated a steeper adaptation of movement speed to curvature. The relationship between movement speed and curvature is further depicted in Fig. 2: raw movement trajectories are plotted, with colour depicting speed, in addition to plots for the relationship between speed and curvature.

*Eq. *1.

1 $$\:v\:\:\propto\:\:\:{\kappa\:\:}^{-\beta\:},\mathrm{\:\:log}\left(v\right)\:\propto\:\:-\beta\:*\:\mathrm{l}\mathrm{o}\mathrm{g}\left(\kappa\:\right)$$

Minimum and maximum speed values were taken as the average of the bottom and top 10% of speed values within a given trial, taken from the log velocity values produced in the speed modulation pre-processing stage.

Spectral Arc Length (SPARC; [88, 89]), a speed-independent measure of movement smoothness, was calculated. For this metric to be comparable between participants, it must be calculated on identical movement trajectories, as SPARC values are higher for longer movement trajectories. SPARC was obtained by calculating the arc length of the magnitude spectrum arising from a Fourier transform of the speed profile. A value for each repeated identical sub-element of each shape within a trial was calculated and values were averaged to obtain a single value per trial.

Finally, an error measure reflecting participants’ deviations from the target shape was calculated for each trial as the absolute mean of the normal distance to the tangent of the nearest point on the shape’s curve [90]. Whilst this provides a broad index of overall tracing accuracy, it should be noted that more subtle, spatially specific variations may be obscured.

Outliers of each kinematic feature, defined as values further than 2 standard deviations away from the mean, were removed: 3.7% of speed, 3.4% of acceleration, 2.1% of jerk, 0.1% of minimum speed, 0.1% of maximum speed, 3.7% of sub-movements, 3.1% of speed modulation, 5.5% of SPARC, 3.7% of error measure. To meet normality assumptions, a log transform was applied to speed, acceleration and jerk values, and a reciprocal transform to the minimum speed values. Variables were then z-scored prior to running linear mixed models.

### Analyses

Group matching was conducted in R Studio (2022.07.2). Linear mixed models (LMMs) were conducted in MATLAB 2022 A using MATLAB’s *fitlme* function. Bayesian analyses and classification models were conducted in JASP (0.17.2.1). Data and analysis scripts are available online at https://osf.io/5g7ft/?view_only=a6bde915a13d46b2ad9c61edaa351260.

#### Group matching

To assess group differences in age, non-verbal reasoning, depression and anxiety, ANOVAs were employed with group as a between-participant factor. Significant differences were further explored using post-hoc t-tests between groups. A chi squared analysis was run to assess group differences in gender. Any variables that were not matched between groups were included in subsequent LMMs as control variables.

#### Group differences

Group differences in parkinsonian traits (UPDRS) and autistic traits (RAADS and AQ) were assessed using ANOVAs with group as a between-participant factor; post-hoc t-tests unpacked any significant group differences.

#### Kinematic differences

To identify kinematic differences between groups, effects-coded LMMs were employed for each kinematic feature with group (ASD, PD, CTRL), shape (clover, petals, ellipse, rounded square), and group-shape interaction as fixed effects. Random effects were included for trial number to account for fatigue or practice effects, day of task administration to account for practice effects, and participant number to account for within-participant similarities across conditions. Group matching variables that were significantly different between groups (i.e., age, depression and anxiety) were also included in the model, and their predictive ability on DV performance was assessed. The model formula was as follows: DV ~ Group * Shape + Age + Depression + Anxiety + (1|Day) + (1|Trial number) + (1|Participant ID). ANOVAs were conducted on the model coefficients to obtain p-values for the fixed effects. Follow-up analyses were conducted for cases in which a main effect of group was observed, or an interaction between group and shape. A different model was used to analyse reaction time data, given there was no ‘shape’ condition: DV ~ Group + Age + Depression + Anxiety + (1|Day) + (1|Participant ID).

To assess evidence for the null hypothesis in cases where group effects were not found, Bayesian ANOVAs were conducted. The input values were the residuals of the DV after controlling for age, anxiety and depression. Fixed factors were group and shape (except in the case of reaction time), and random factors were participant ID, trial number (except in the case of reaction time) and day. Bayes Factors (BF_01_) for the main effect of group are reported, with values of 3–10 and 1–3 taken as moderate and anecdotal evidence for the null hypothesis respectively [91].

#### Classification analyses

##### Methods of classification

To identify how well the three groups could be classified using the obtained data, we employed three methods of classification: K-Nearest Neighbours (KNN), Random Forest (RF), and Support Vector Machine (SVM). These three classification methods have been widely used alongside each other in studies of similar sample sizes classifying parkinsonian movement [32–40]. Each classification algorithm was based on data from 80% of the participants and the test accuracy relates to the accuracy of the classification algorithm in classifying the remaining 20% of the participants. Test accuracy is reported for each method, as well as the mean test accuracy across all three methods. The number of nearest neighbours (KNN) and decision trees (RF) were optimised for each analysis as per JASP functionality, and the SVM classifications employed a linear kernel.

##### Feature selection

Input variables for the analyses consisted of kinematic features (two sets: *all kinematic features* and *discriminatory kinematic features*) and questionnaire measures (three sets: *ASD*, *PD*, and *ASD & PD*).

To extract *all kinematic features*, data from each participant was used to calculate a single value of each kinematic feature for each of the four shapes: residuals for all DVs were calculated after controlling for age, depression and anxiety, and an average was taken for data from the same shape across trials and days where relevant. This resulted in 37 input variables for the *all kinematic features* dataset (four shapes x nine kinematic features, plus one reaction time value per participant averaged across days).

*Discriminatory kinematic features* were identified using a supervised filter-based feature selection in which kinematic features were selected according to group difference significance. For the ASD versus PD classification, DVs that were significantly different between ASD and PD in the above LMM analyses (*p* <.05) were selected. If a main effect of group was found for a given DV between the two groups, then values for that DV on all shapes would be included in the *discriminatory kinematic features* dataset. However, if a group by shape interaction was observed which only yielded a group difference for a particular shape, then the DV value for that specific shape was included in the *discriminatory kinematic features* dataset. When multiple groups were included in a classification model (as in the clinical versus non-clinical classification), *discriminatory kinematic features* included all DVs that significantly differed across the two sides of the classification model: DVs that significantly differed between ASD and CTRLs, and PD and CTRLs, were included, but not those that significantly differed between ASD and PD (as they grouped as the same class).

*ASD questionnaires* comprised performance on the AQ and RAADS, and the *PD questionnaire* comprised performance on the UPDRS. All three questionnaires were included in the *ASD & PD questionnaires* set. Scores used in the classification analyses were residuals after controlling for age, depression and anxiety.

##### Model details

We investigated whether kinematic features and/or questionnaires were able to classify whether an individual belonged to a clinical or non-clinical population, and subsequently which clinical group an individual belonged to (ASD or PD). For the former, the ASD and PD groups were coded as 1, and the CTRL group as 0, and classification was assessed for ASD/PD versus CTRLs. For the latter, analyses were run just on the ASD and PD participants and classification was assessed for the ASD versus PD comparison. Eleven versions of the group classification analyses were run, comprising the different combinations of the questionnaires and kinematic features.

### Pre-Registration

A pre-registration for the study can be found online at https://osf.io/dnk6j. Contrary to the pre-registration, here data is only analysed from participants with PD OFF their dopaminergic medication and not ON medication. This was to ensure that movement in the PD group was not influenced by medication and was thus a more accurate representation of how they might present pre-diagnosis. Following discussions with collaborators post pre-registration, questionnaires were added as control variables (PHQ and GAD). All participants also completed the UPDRS as a measure of parkinsonian traits, as well as the RAADS as an additional measure of autistic traits. Additional kinematic features were calculated beyond the pre-registered parameters of speed, acceleration, jerk, sub-movements and speed modulation values (i.e., SPARC, error measure, minimum speed, maximum speed, and reaction time). Finally, classification analyses, listed as exploratory analyses in the pre-registration, are reported.

## Results

### Group matching

Table 1 presents descriptive statistics for all groups, in addition to tests of equivalence (see Appendix 2 (Table A2) for ethnicity information). The three groups were matched on gender and non-verbal reasoning ability. Whilst both clinical groups were age-matched to the CTRL group (ASD-CTRL: *p >*.05; PD-CTRL: *p >*.05), the ASD and PD groups were not age-matched (*t*(60.57)=−3.88, *p* <.001). Additionally, the CTRL group had significantly lower levels of depression compared to both the ASD group (*t*(42.26) = 4.67, *p* <.001) and the PD group (*t*(45.54) = 5.11, *p* <.001), and the ASD group had significantly higher levels of anxiety than both the CTRL group (*t*(58.08) = 4.57, *p* <.001) and the PD group (*t*(55.42) = 3.64, *p =*.001). As such, age, depression and anxiety were included in subsequent models as covariates.

**Table 1:** Descriptive statistics and tests of equivalence for the autism, parkinson’s disease and control groups

	ASD	PD	CTRL	Test of equivalence	Group differences
Gender	14 M, 16 F, 1 O	19 M, 13 F	15 M, 16 F	*X*^*2*^(4) = 3.25	
Age	55.52[8.01]	63.16[7.60]	59.00[9.96]	*F*(2, 91) = 6.27**	PD > ASD***
Non-verbal reasoning	0.57[0.15]	0.55[0.13]	0.58[0.16]	*F*(2, 89) = 0.43	
Depression	8.2[5.59]	7.87[4.56]	3.0[2.58]	*F*(2, 89) = 13.26***	ASD > CTRL***; PD > CTRL***
Anxiety	9.9[5.89]	5.1[4.39]	3.6[4.90]	*F*(2, 89) = 12.86***	ASD > CTRL***; ASD > PD**
Years since diagnosis	4.23[2.69]	3.69[2.38]	-	-	
PD traits (UPDRS)	3.7[3.76]	11.91[6.93]	0.94[1.69]	*F*(2, 91) = 46.81***	PD > CTRL***; PD > ASD***; ASD > CTRL***
Autistic traits (RAADS)	32.97[8.05]	10.7[9.43]	6.32[6.46]	*F*(2, 89) = 97.31***	ASD > CTRL***; ASD > PD***; PD > CTRL*
Autistic traits (AQ)	37.61[7.06]	19.19[7.72]	16.03[5.95]	*F*(2, 91) = 87.19***	ASD > CTRL***; ASD > PD***

Table contains means (M) and standard deviations (SD): M[SD]. Significant tests of equivalence indicate differences between groups. Group differences reflect results of t-test group comparisons. ASD = Autism Spectrum Disorder, PD = Parkinson’s Disease, CTRL = Control, M = male, F = female, O = other. *** *p* <.001, ** *p* <.01 and * *p* <.05

### Group differences

As expected, the PD group had significantly higher levels of PD traits, as measured via the UPDRS, than both the CTRL (*t*(34.80) = 8.70, *p* <.001) and ASD (*t*(48.13) = 5.84, *p* <.001) groups. Interestingly, the ASD group had elevated PD traits compared to the CTRL group (*t*(41.67) = 3.79, *p* <.001), evidencing similarities in the characteristics of ASD and PD. An item analysis revealed that the ASD group scored higher than CTRLs on a wide range of motor function domains (see Appendix 4). The ASD group had significantly higher autistic traits than both the CTRL group (RAADS: *t*(57.30) = 14.37, *p <*.001; AQ: *t*(58.32) = 13.01, *p* <.001) and PD group (RAADS: *t*(56.96) = 9.91, *p* <.001, AQ: *t*(60.81) = 9.89; *p <*.001). The PD group had elevated ASD traits compared to the CTRL group on the RAADS (*t*(51.13) = 2.11, *p* =.040) but not the AQ (*t*(58.10) = 1.82, *p* =.074), again providing evidence for similarities between the two conditions. An item analysis indicated that this effect was driven by items indexing barriers to social interaction (see Appendix 5).

### Kinematic differences

#### Groups can be distinguished by kinematic features

The ASD group exhibited differences in speed modulation compared to the PD and CTRL group. A significant interaction between group and shape was observed in the main LMM (*F*(6, 4482) = 13.37, *p* <.001). For the ASD and PD comparison, a significant interaction between group and shape (*F*(3, 2655) = 15.81, *p* <.001) was unpacked to reveal a significant difference between the two groups for speed modulation values on the rounded square (*F*(1, 658) = 5.40, *p =*.020), in which values in the ASD group were higher than those in the PD group (beta estimate = 0.229, 95% CI [0.035, 0.423]). This result was also reflected in the ASD and CTRL comparison, in which the exploration of a significant interaction between group and shape (*F*(3, 3601) = 10.91, *p* <.001), revealed a significant difference between groups for the rounded square (*F*(1, 898) = 5.92, *p* =.015); ASD values were higher than the CTRL group (beta estimate = 0.216, 95% CI [0.042, 0.390]). A significant group by shape interaction between PD and CTRL (*F*(3, 2705) = 13.86, *p* <.001) did not yield significant group effects for any individual shape (all *p* >.05).

The PD group and CTRL group also differed from the ASD group in terms of sub-movements. A significant interaction between group and shape was present in the main LMM (*F*(6, 4450) = 3.75, *p* <.001). When comparing ASD and PD groups, a main effect of group was observed (*F*(1, 2637) = 4.46, *p* =.035), whereby a greater number of sub-movements were used by the ASD group (beta estimate = 0.197, 95% CI [0.014, 0.379]). A significant interaction between group and shape was also observed (*F*(3, 2637) = 5.55, *p* <.001). Further analyses revealed a main effect of group for the clover (*F*(1, 694) = 7.76, *p* =.005; beta estimate = 0.151, 95% CI [0.045, 0.258]), petals (*F*(1, 680) = 5.067, *p =*.025; beta estimate = 0.217, 95% CI [0.028, 0.405]), and the rounded square (*F*(1, 662) = 6.12, *p* =.014; beta estimate = 0.288, 95% CI [0.059, 0.517]); in each case, an increased number of sub-movements were used by the ASD group compared to the PD group. For the ASD and CTRL comparison, a main effect of group was observed (*F*(1, 3544) = 4.00, *p* =.046), with a greater number of sub-movements in the ASD group (beta estimate = 0.181, 95% CI [0.004, 0.359]). A significant group by shape interaction (*F*(3, 3544) = 4.14, *p* =.006) was unpacked to reveal a significant main effect of group for the clover (*F*(1, 933) = 6.42, *p* =.011; beta estimate = 0.127, 95% CI [0.029, 0.226]), petals (*F*(1, 905) = 4.00, *p* =.046; beta estimate = 0.177, 95% CI [0.003, 0.350]), and the rounded square (*F*(1, 898) = 5.46, *p* =.020; beta estimate = 0.267, 95% CI [0.043, 0.491]).

For the reaction time data, a main effect of group was observed in the main LMM (*F*(2, 140) = 3.90, *p* =.022). Further analyses revealed that the only significant group difference in reaction time was between the PD group and ASD group (*F* [1, 83] = 7.18, *p* =.009); ASD reaction times were shorter than PD reaction times (beta estimate=−0.023, 95% CI [−0.040, −0.006]).

**Fig. 3:**
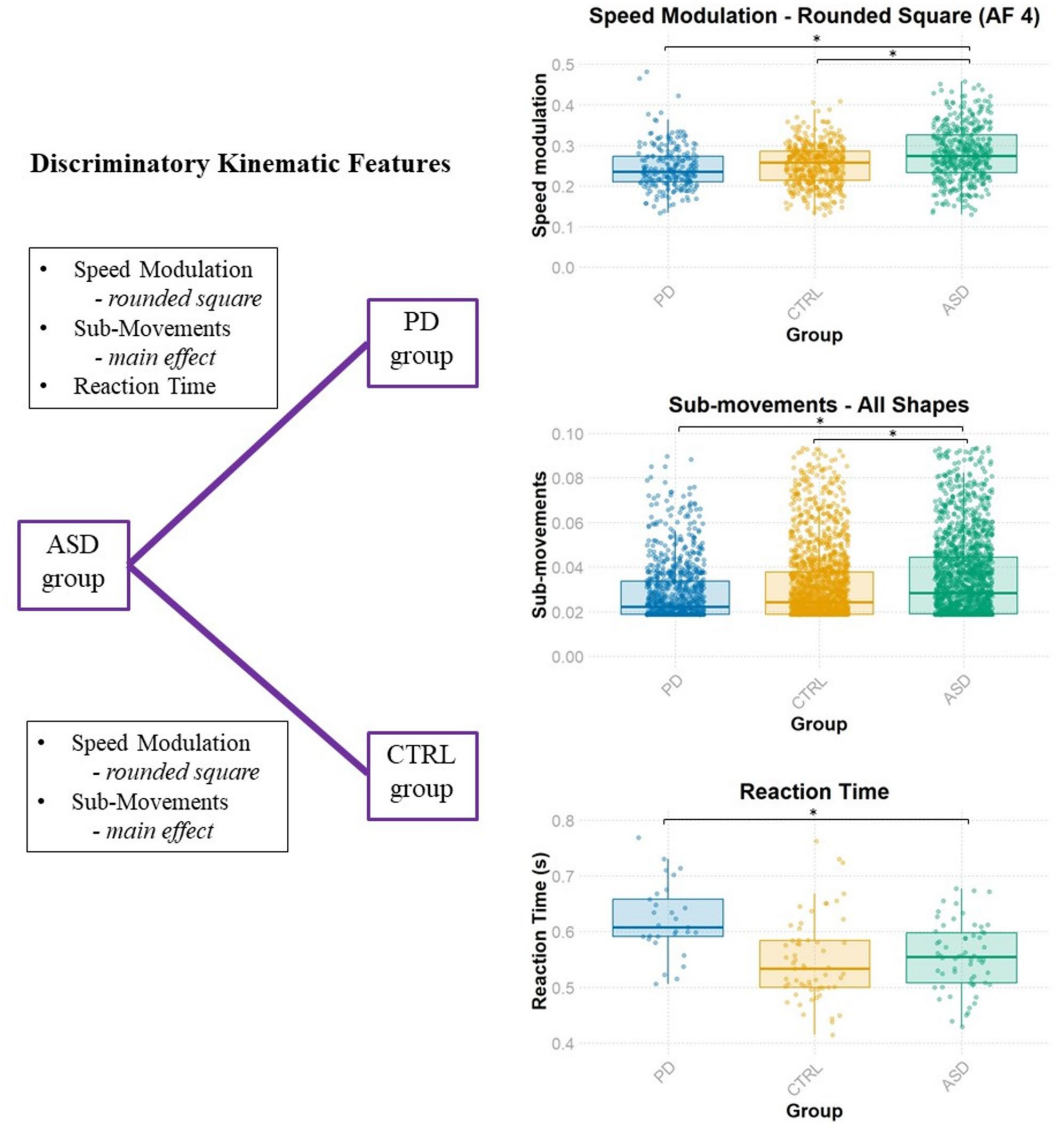
The Discriminatory Kinematic Features Identified for Each Group Comparison. Note. The left figure lists the discriminatory kinematic features determined by a series of linear mixed models, in which a significant main effect of group (either overall or for a given shape) led to the incorporation of that dependent variable as a discriminatory kinematic feature for that group comparison. The right figures depict group differences in the discriminatory kinematic features. The speed modulation box plot (*top*) contains one data point per trial, including only trials for the rounded square. The sub-movement box plot (*middle*) contains one data point per trial, including data for all shapes. The reaction time plot (*bottom*) includes one data point per participant (average reaction time across all trials). Each box plot depicts the median and interquartile range (IQR), with 1.5 x IQR as whiskers. AF = Angular Frequency; ASD = Autism Spectrum Disorder (green); CTRL = Control (orange); PD = Parkinson’s Disease (blue). * denotes significant difference between groups.

In sum, the ASD group significantly differed from the CTRL group in terms of speed modulation values for the rounded square and sub-movement values (main effect). By contrast, reaction time data was able to distinguish the PD and ASD groups, as well as speed modulation values for the rounded square and sub-movement values (main effect). These DVs were used to determine the *discriminatory kinematic features* in subsequent classification models (Fig. 3). Box plots depicting raw data for all variables, separately for groups (ASD, PD, CTRL) and shapes (clover, petals, ellipse, rounded square), can be found in Appendix 7 (Figures A1-A9).

#### Some kinematic features are the same between groups

No group differences were found for speed, acceleration, jerk, SPARC, or maximum and minimum speed (whilst some group by shape interactions were significant, *p* >.05 for main effects of group all post-hoc analyses). Subsequently, Bayesian ANOVAs were conducted on each of these kinematic features, with the main effect of group being the result of interest. Moderate evidence for the null hypothesis of no differences between groups, as indicated by Bayes Factors (BF_01_) of 3–10, was found for SPARC (BF_01_ = 5.987) and maximum speed (BF_01_ = 8.131). Anecdotal evidence for the null hypothesis, as indicated by Bayes Factors (BF_01_) of 1–3, was found for speed (BF_01_ = 1.490), acceleration (BF_01_ = 2.165), jerk (BF_01_ = 2.671), and minimum speed (BF_01_ = 2.852). BF_01_ values for the main effect of group for each group comparison are reported in Appendix 3 (Table A3).

Importantly, the groups did not differ with respect to the error measure (*p* >.05), thus providing evidence that any kinematic differences did not arise from significant deviations in the spatial location of the stylus tip when tracing the shapes. This was supported by moderate evidence for the null hypothesis as indicated by a BF_01_ value of 4.301.

As noted in the analysis above, no differences were found between the PD group and the CTRL group in terms of speed modulation, sub-movements, or reaction time (all *p* >.05). This was supported by moderate evidence for the null hypothesis for speed modulation (BF_01=_4.071), and anecdotal evidence for sub-movements (BF_01=_1.951) and reaction time (BF_01=_1.709). Further to this, the lack of group difference between the ASD group and the CTRL group for reaction time (*p* >.05) was supported by moderate evidence for the null hypothesis (BF_01=_4.974).

#### Age is a predictor of kinematic features, but anxiety and depression are not

Age significantly predicted speed modulation values (*F*(1, 4482) = 6.06, *p* =.014), whereby speed modulation values decreased as age increased (beta estimate=−0.017, 95% CI [−0.030, −0.003]). A negative prediction was also observed for sub-movements (*F*(1, 4450) = 13.02, *p* <.001; beta estimate=−0.025, 95% CI [−0.039, −0.012]). Slower reaction times were observed as age increased (*F*(1, 140) = 9.18, *p* =.003; beta estimate = 0.002, 95% CI [0.001, 0.004]). Finally, age was also a significant negative predictor of speed (*F*(1, 4450) = 13.20, *p* <.001; beta estimate=−0.037, 95% CI [−0.056, −0.017]), acceleration (*F*(1, 4464) = 8.13, *p* =.004; beta estimate=−0.029, 95% CI [−0.049, −0.009]), and jerk (*F*(1, 4527) = 5.19, *p* =.023; beta estimate=−0.023, 95% CI [−0.043, −0.003]). No significant predictions of age were observed for SPARC, error measure, minimum speed or maximum speed (all *p >*.05). Depression and anxiety did not predict any of the kinematic features (all *p* >.05).

### Classification analyses

**Fig. 4:**
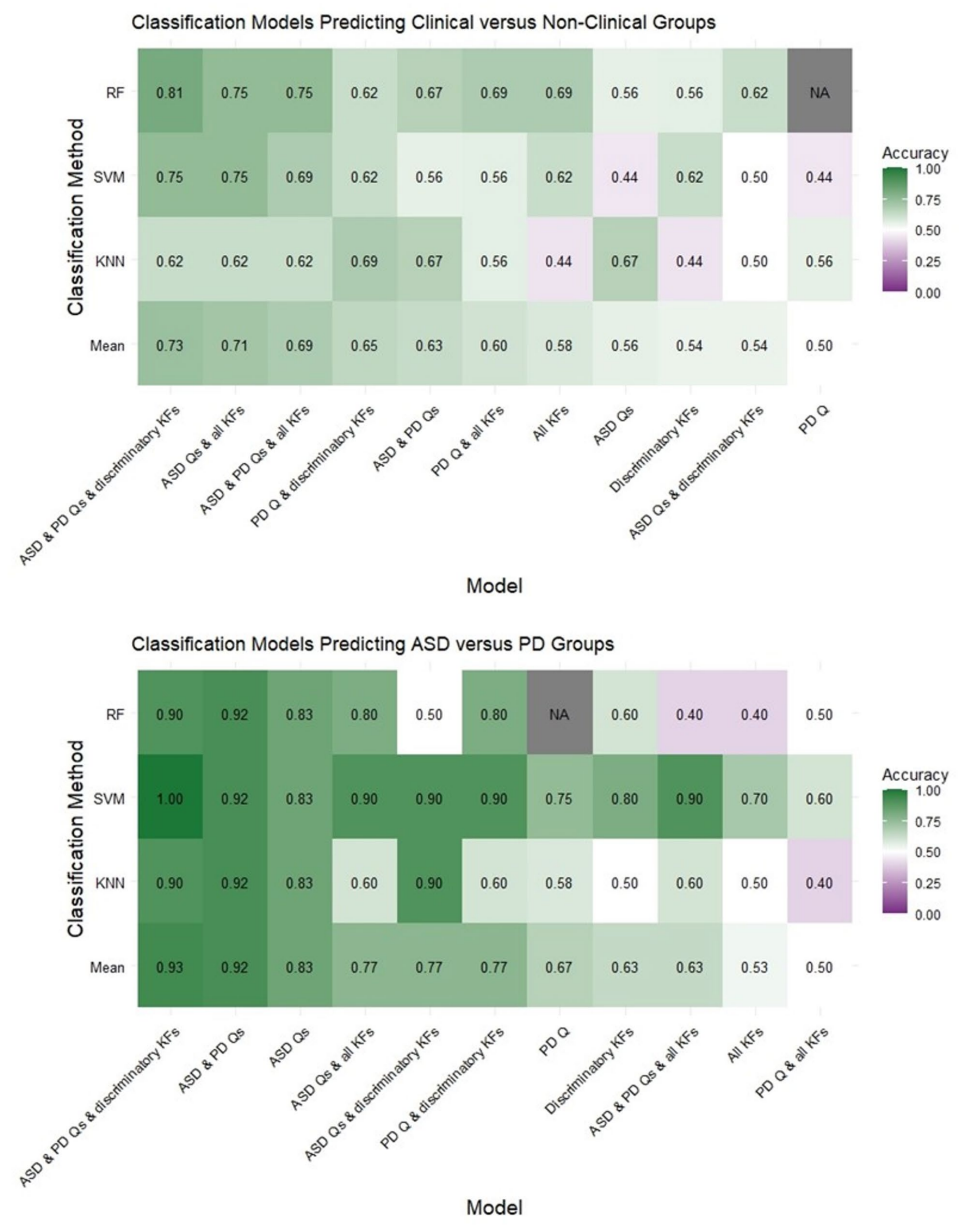
Test accuracy scores for all classification models. Note. Heat maps represents test accuracy from the clinical versus non-clinical group classification (*top*) and the ASD versus PD group classification (*bottom*). Different rows represent test accuracy scores for each of the classification methods (KNN, SVM, RF) and the mean of the three methods. Different columns represent the different combinations of predictors used for each classification model, and are ordered from left to right based on descending mean test accuracy. Cells contain their test accuracy score and are colour-coded whereby test accuracy scores above chance are green and those below chance are purple. ASD = Autism Spectrum Disorder; KF = Kinematic Features; KNN = K-Nearest Neighbours; PD = Parkinson’s Disease; Q = Questionnaire; RF = Random Forest; SVM = Support Vector Machine.

When classifying the ASD, PD and CTRL groups into clinical (i.e., ASD and PD) and non-clinical (CTRL) groups, the highest mean test accuracy was obtained from a model combining discriminatory kinematic features and ASD & PD questionnaires (mean test accuracy = 0.73). This was similarly the case when classifying the ASD versus PD groups (mean test accuracy = 0.93), though some specific classification methods also demonstrated high utility of kinematics alone (e.g., discriminatory kinematic features SVM test accuracy = 0.80). For classifications involving questionnaires, using both ASD and PD questionnaires typically yielded higher mean test accuracy scores than the individual questionnaires alone. Variability was seen in whether discriminatory kinematic features or all kinematic features yielded stronger classification accuracy scores. The mean test accuracy for all models was above chance, except for two models performing at chance (mean test accuracy = 0.5); accuracy scores for all classification models are depicted in Fig. 4.

## Discussion

In the current study, we set out to identify both similarities and differences in autistic and parkinsonian movement. A number of kinematic features did not differ between autistic individuals and individuals with PD. However, these features were not found to be distinct from the general population. Confirmatory evidence of kinematic similarity would have comprised movement features that are similar between ASD and PD but differ from the general population. Thus, our approach did not identify a kinematic similarity explanation for an apparent likeness between movement profiles of autism and PD.

Turning to movement differences, it appears that a number of kinematic features could be used to *distinguish* between the three groups: autistic movement differed from parkinsonian movement in terms of speed modulation values, sub-movements, and reaction time, and only differed from the general population with respect to speed modulation values and sub-movements. Thus, combinations of these kinematic features may be useful for distinguishing the three groups. These findings are in line with extant literature highlighting steeper speed modulation values and a greater number of sub-movements in autism [48, 62, 63], and slower reaction times in PD [60]. These observed differences in kinematic features may reflect distinct underlying neuro-cognitive mechanisms of motor control. For example, the increased number of sub-movements in the ASD group is consistent with the ‘weak central coherence’ hypothesis [62], whereby a focus on local rather than global information leads to more corrective micro-adjustments during movement. Similarly, altered speed modulation may reflect differences in anticipatory planning, (i.e., adapting speed based on the tightness of the upcoming trajectory). In contrast, slower reaction times in PD may relate to difficulties in movement initiation, particularly given the absence of group differences in speed itself in the Shapes Tracing Task. Nevertheless, the current study cannot disambiguate between potential neurocognitive explanations and further studies specifically designed for this purpose are necessary.

As posited in the Introduction, dopamine is a plausible neurobiological substrate underlying these movement differences. Indeed, previous work indicates high dopaminergic states lead to increased speed modulation [92]. Given that a hyper-dopaminergic state has been linked to autism [26], our finding of increased speed modulation in autism is consistent with these findings. By contrast, PD is characterised by a hypo-dopaminergic state, meaning we would have expected to see lower speed modulation values in PD compared to the control group; this was not the case. In addition, no differences between the PD and control group in speed were observed, a kinematic marker previously associated with dopamine [92]. One explanation for this discrepancy is that within-participant manipulations of dopamine, as used in Hickman and colleagues (e.g., ON vs. OFF medication, or placebo vs. haloperidol), create larger shifts in dopamine than naturally occurring differences between PD-OFF and control groups. Indeed, dopaminergic medication can sometimes push individuals into a hyper-dopaminergic state depending on baseline levels, a phenomenon described as the ‘dopamine overdose hypothesis’ [93, 94]. In our study, although PD participants completed the task OFF-medication, residual dopaminergic effects from prior doses may have persisted, further reducing differences from controls.

Despite the presence of kinematic differences between groups, these were not seen for all shapes in the Shapes Tracing Task. This is consistent with findings that differences in kinematic features are context-dependent [95]. Most notably, the ellipse was the *least* helpful for distinguishing groups. This is particularly important given that many drawing tasks in the literature only index elliptical or spiral movements [32, 37, 40, 63, 96], with only one other study utilising other angular frequency-defined shapes [62]. As such, we recommend that future studies employ a range of shapes from across the full angular frequency spectrum to observe the strongest group differences in kinematic features.

A number of kinematic variables did not differ between groups, which in some cases were in conflict with previous literature. For example, some components of Cook and colleagues’ [62] study were not replicated here, including increased maximum speed in autism. In addition, we did not support existing findings that autistic movement is characterised by increased speed, acceleration and jerk [44, 47]. However, low Bayes factors were observed for the group similarities on these variables, indicating that there is not strong evidence to conclude that movements were the same between groups. There are a number of possible explanations for the lack of group differences. Task-specific constraints such as the resistive forces of the stylus on the tablet and limitations in sampling rate or spatial resolution may have reduced group differences, in contrast to motion tracking tasks that do not require the use of a tablet and stylus [44, 52]. In addition, movement kinematics are highly context-dependent. Recent work using tablet-based tasks in autistic children has shown that kinematic features vary across tasks [95], meaning our pattern of results may be specific to the task used. Importantly, unexpected findings in the autism group may be because our sample comprised older autistic adults, a group in which kinematic features have not yet been assessed. For instance, evidence suggesting jerky movements in autism has come from younger samples of autistic individuals (e.g., Cook et al., 2013 [44]; mean age = 41.07) or children (e.g., Grace et al., 2017 [47]; mean age = 10.58). It may be the case that jerk differences between autistic and non-autistic individuals are not present in older age, perhaps due to a change in kinematic features in the non-autistic population. This pattern of results may be accounted for by the accelerated aging theory of autism [97], which proposes that autistic individuals may show earlier onset of age-related changes. Differences in kinematic features between autistic and non-autistic individuals may be more apparent at younger ages, but become less pronounced in later life as age-related changes in the non-autistic population progress.

Further to the above explanations, our data may provide unique insight into the theory of decreased movement smoothness in autism [44, 47, 62, 98]. Whilst an increased number of sub-movements were observed in the ASD group, there were no significant differences in jerk and SPARC. However, an interesting distinction can be made between these two measures: evidence for no difference between ASD and CTRL was twice as strong for SPARC compared to jerk. Given that SPARC is a speed-independent measure of movement smoothness, whereas jerk is not, this leads us to question whether previous evidence showing increased jerk in autism might in fact reflect an epiphenomenal consequence of a difference in movement speed; in other words, a true group difference in movement speed may cause an apparent difference in jerk due to the fact that the calculations of the two kinematic features are inherently linked.

Another core aim of the study was to assess the utility of kinematic measures in predicting clinical groups instead of, or in addition to, questionnaire measures. Combining kinematic features and questionnaire measures yielded the strongest classification accuracy for both classifications (clinical versus non-clinical populations, and ASD versus PD populations). A classification algorithm that can distinguish between clinical and non-clinical populations is particularly important given that we demonstrated changes to kinematic features with increasing age: there is a need to distinguish movement decline arising from clinical conditions as opposed to normal ageing. Given the utility of kinematic features in conjunction with questionnaires to distinguish between these groups, future work should investigate their utility for early screening of clinical conditions in conjunction with clinical assessments. In the case of ASD versus PD, kinematics were also useful in other models which utilised particular classification methods, both alone and in conjunction with questionnaires. This indicates that, once it has been determined that an individual exhibits kinematic features that differ from normal ageing, further examination of these kinematic features can reveal which clinical group the individual belongs to. It should be noted that the models in which kinematics were useful were not always those containing only the *discriminatory kinematic features* (i.e., those that were found to significantly differ between groups). Certain classifications yielded stronger accuracy when using *all kinematic features* as opposed to *discriminatory kinematic features*. Thus, variables that do not significantly differ between groups may be useful for distinguishing groups in a classification algorithm in conjunction with other variables. Following this demonstration of the utility of movement kinematics in classifying autism and PD, future studies should recruit a larger and more diverse sample to develop an algorithm with clinical potential. This could be implemented alongside existing PD assessments to improve the specificity of the diagnostic process.

A move towards including kinematic features in clinical screening tools has advantages over self-report measures. Kinematic features are not susceptible to social desirability bias and camouflaging, unlike autism questionnaires [99–101]. Arm movement, specifically, may be useful given that more socially-relevant movements such as facial expressions may be altered due to camouflaging behaviours [102, 103]. With respect to inaccurate self-report, co-occurring conditions such as alexithymia, a difficulty identifying and describing one’s own emotions [104–106], may lead to difficulties introspecting for the purposes of questionnaire completion [107, 108]. This may be particularly exacerbated in autism questionnaires which often refer to thoughts and feelings regarding one’s emotions, and it is notable that elevated alexithymic traits have been observed in both autistic [109, 110] and PD [111, 112] populations. Similarly, minimally verbal individuals may struggle to complete self-report measures, which is an important concern as these individuals are thought to make up 25–35% of the autistic community [113]. Moving on to self-reported movement differences, subjective questions about symptom severity can yield inaccurate responses due to individual differences in the interpretation of categories (i.e., “mild” versus “severe”). Clinician-based assessments are also limited by intra- and inter-rater variability, and calls for more objective movement-based analyses have been made [114]. Our study involved a remote assessment of movement kinematics using a touch-screen device, something which could be readily implemented in large-scale or community-based studies, reducing the need for in-person laboratory or clinical kinematic assessments. Overall, refining objective measures of autistic and parkinsonian traits, such as kinematic features, has potential for advancing diagnostic accuracy.

## Limitations

The current study has a number of limitations. Firstly, to ensure specificity of ASD and PD groups, all autistic participants were required to *not* have a diagnosis of PD and vice versa. Control participants likewise reported having no diagnosis of either condition. However, we acknowledge that a lack of diagnosis does not necessarily exclude the presence of undiagnosed cases, and without a longitudinal design it is not possible to know whether any of the participants in the ASD group may later go on to develop PD. In addition, the PD and CTRL participants come from a generation in which ASD diagnoses were not prevalent [73]; indeed, the autistic participants in our sample only received their diagnoses a mean of 4.23 years prior to completing the study. Future studies may benefit from conducting formal ASD and PD assessments on all participants, or adopting longitudinal designs, to confirm diagnostic status more robustly.

Secondly, participants in our PD sample had relatively recent diagnoses and did not have extreme tremor (which would have interfered with task completion). This sample selection may explain why significant differences were not found between PD and CTRL groups. Importantly, Bayes factors for the null group differences between PD and CTRLs only revealed anecdotal evidence, meaning it cannot be strongly concluded that kinematics were the same between groups.

Thirdly, our remote testing protocol, made necessary due to the COVID-19 pandemic, resulted in limitations to the data we could collect. For example, we had limited information on the clinical characteristics of the PD group, beyond years since diagnosis and the UPDRS self-report scale, due to the inability to conduct thorough in-person assessments. In addition, touch-screen tablets were used to facilitate remote data collection, something which also allowed us to demonstrate the feasibility of widespread, low-cost deployment of the task. However, compared to in-person devices with a higher sampling rate, it is possible that a degree of experimental sensitivity was lost. This may have limited our ability to strongly conclude a lack of differences between groups, though it speaks to the robustness of the significant differences that were uncovered.

Fourthly, limitations in sample diversity should be highlighted, including a high proportion of White-British participants and an absence of minimally verbal autistic individuals. The recruitment of a more diverse sample in future research could help refine classification models: recent classification models of PD and non-PD handwriting have developed sex-specific and age-dependent models for optimal classification [42, 115].

Finally, whilst the current study focused on fine motor function, future work should compare groups on gross motor function tasks, which are commonly used in PD diagnostic processes.

## Conclusions

To summarise, in the current study we identified both similarities and differences in the movement profiles of autistic individuals, individuals with PD, and the general population in a task of restricted movement. Whilst the three groups did not differ on a range of kinematic features, points of distinction between the three groups included speed modulation values, sub-movements and reaction time. We therefore propose that kinematic features may help distinguish between the three groups. In a number of classification algorithms, kinematic features (either alone or in conjunction with questionnaire measures) yielded strong classification accuracy in models predicting the three participant groups (i.e., when distinguishing clinical groups from the general population and subsequently identifying which clinical group an individual belonged to). Future work should therefore investigate the utility of kinematic features in the development of more selective diagnostic procedures.

## Supplementary Information

Supplementary Material 1.

## Data Availability

Data and analysis scripts are available online at https://osf.io/5g7ft.
