# Supplementary material for: An assessment of autistic and parkinsonian movement profiles to inform selective classification algorithms
Source: J Neurodev Disord. 2026 Jan 10;18:8. doi: 10.1186/s11689-025-09668-8 (PMC12882580; doi:10.1186/s11689-025-09668-8)
Supplement: Supplementary file 1 — Supplementary Material 1. [file 11689_2025_9668_MOESM1_ESM.docx]

**Appendix 1**

**Table A1**

*Types of PD Medications Taken by Participants in the PD group*

|  | **N** |
| --- | --- |
| **Combined Totals** |  |
| Levodopa | 23 |
| Dopamine Agonists | 16 |
| MAO Inhibitors | 10 |
| **Those Taking Only One Type of Medication** |  |
| Levodopa | 9 |
| Dopamine Agonists | 5 |
| MAO Inhibitors | 2 |
| **Those Taking Two Types of Medications** |  |
| Levodopa & Dopamine Agonists | 11 |
| Levodopa & MAO Inhibitors | 2 |
| Dopamine Agonists & MAO Inhibitors | 1 |
| **Those Taking Three Types of Medications** |  |
| Levodopa, Dopamine Agonists & MAO Inhibitors | 2 |

*Note*. Combined totals refer to how many participants were taking each type of medication regardless of the other medications being taken. Subsequent rows break down participants into those taking only one type of medication, two types of medication, or all three types of medication. It should be noted that participants completed the task before their first dose of medication in the morning. MAO = Monoamine oxidase.

**Appendix 2**

**Table A2**

*Ethnicity Information for the Autism, Parkinson’s Disease and Control Groups*

|  | **ASD** | **PD** | **CTRL** | **Total** |
| --- | --- | --- | --- | --- |
| Asian Pakistani | 0 | 0 | 4 | 4 |
| White & Pakistani | 0 | 0 | 1 | 1 |
| White American | 0 | 1 | 0 | 1 |
| White British | 28 | 29 | 26 | 83 |
| White European | 3 | 0 | 0 | 3 |
| White Irish | 0 | 1 | 0 | 1 |
| White Other | 0 | 1 | 0 | 1 |

*Note.* Table contains count data for each cell. ASD = Autism Spectrum Disorder; PD = Parkinson’s Disease; CTRL = Control.

**Appendix 3**

**Table A3**

*Bayes Factors for Kinematic Features with No Group Differences*

|  | **All groups** | **ASD-CTRL** | **ASD-PD** | **PD-CTRL** |
| --- | --- | --- | --- | --- |
| Maximum Speed | 8.131 | 3.046 | 6.120 | 2.124 |
| SPARC | 5.987 | 6.088 | 2.840 | 1.421 |
| Error Measure | 4.301 | 2.451 | 1.861 | 1.361 |
| Minimum Speed | 2.852 | 0.792 | 4.652 | 2.751 |
| Jerk | 2.671 | 3.421 | 1.935 | 1.608 |
| Acceleration | 2.165 | 2.550 | 1.225 | 1.734 |
| Speed | 1.490 | 1.869 | 0.788 | 1.560 |

*Note.* Table contains BF_01_ values for the main effect of group obtained in Bayesian ANOVAs. ASD = Autism Spectrum Disorder, PD = Parkinson’s Disease, CTRL = Control, SPARC = Spectral Arc Length.

**Appendix 4**

**Item analysis of the UPDRS between ASD and CTRL**

In the main text, it is noted that the ASD group have elevated PD traits compared to the CTRL group as indexed by the UPDRS. We subsequently conducted t-tests for each item on the UPDRS. The ASD group had significantly higher scores than the CTRL group on 8 out of the 13 items:

(1) *Over the past week, have you had problems with your speech?* (*t*(33.26) = 3.18, *p* = .003);

(2) *Over the past week, have you usually had problems swallowing pills or eating meals?* (*t*(39.53) = 2.62, *p* = .012);

(3) *Over the past week, have you usually had troubles handling your food and using eating utensils? For example, do you have trouble handling finger foods or using forks, knives, spoons, chopsticks?* (*t*(30) = 2.53, *p* = .017);

(4) *Over the past week, have you usually had problems dressing? For example, are you slow or do you need help with buttoning, using zippers, putting on or taking off your clothes or jewelry?* (*t*(30) = 2.40, *p* = .023);

(5) *Over the past week, have you usually been slow or do you need help with washing, bathing, shaving, brushing teeth, combing your hair, or with other personal hygiene?* (*t*(30) = 2.11, *p* = .043);

(6) *Over the past week, have people usually had trouble reading your handwriting?* (*t*(43.48) = 2.20, *p* = .033);

(7) *Over the past week, have you usually had trouble doing your hobbies or other things that you like to do?* (*t*(32.77) = 3.03, *p* = .005);

(8) Over the past week, have you usually had problems with balance and walking? (*t*(37.21) = 2.31, *p* = .027).

These items span a wide variety of PD traits, including both fine and gross motor function. The remaining 4 items that were *not* significantly different between ASD and CTRLs refer to too saliva in the mouth, trouble turning in bed, difficulty getting out of a deep seat, shaking/tremor, and sudden freezing when walking. In sum, the ASD group’s elevated PD traits reflected differences on a wide range of movement and motor function domains.

**Appendix 5**

**Item analysis of the RAADS between PD and CTRL**

It is noted in the main text that the PD group have elevated autistic traits compared to the CTRL group as indexed by the RAADS. The PD group had significantly higher scores than the CTRL group on 3 out of the 14 items:

(1) *It is very difficult for me to work and function in groups.* (*t*(51.56) = 2.17, *p* = .035);

(2) *How to make friends and socialize is a mystery to me.* (*t*(38.02) = 2.75, *p* = .009);

(3) *When talking to someone, I have a hard time telling when it is my turn to talk or to listen.* (*t*(44.36) = 2.60, *p* = .013).

Each of these items can be interpreted in the context of physical barriers to social situations. Items relating to the understanding of social situations (e.g., understanding how others are feeling and what they expect) were not significantly different between groups. Other non-significant items included being overwhelmed by senses, focusing on detail, taking things too literally, and difficulty with change. Overall, it appears that elevated autistic traits in the PD group are driven by an inability to become involved in social situations.

**Appendix 6**

**Day-matching exploratory analyses**

Analyses in main text include data from two days for the ASD and CTRL groups and one day for the PD group (N = 14 for day 1 and N = 18 for day 2). To ensure that this distribution of data did not impact the results, we conducted exploratory analyses in which data from one day were randomly selected for participants in the ASD and CTRL groups, match the proportions of day 1 / day 2 data in the PD group. Group difference analyses were then re-run using this data subset. All previous significant results remained significant in these analyses. One new significant result emerged. As in main text, a significant interaction between group and shape for the ASD-CTRL comparison of speed modulation was found. However, a significant main effect of group was also present (*F*(1,1775) = 4.06, *p* = .044). Importantly, as is the case in main text, there was only a significant group difference for the rounded square (*F*(1,434) = 4.18, *p* = .042; main effect of group for all other shapes *p* > .05), meaning results did not substantively change.

**Appendix 7**

*Figure A1. Speed values for all trials across groups and shapes*


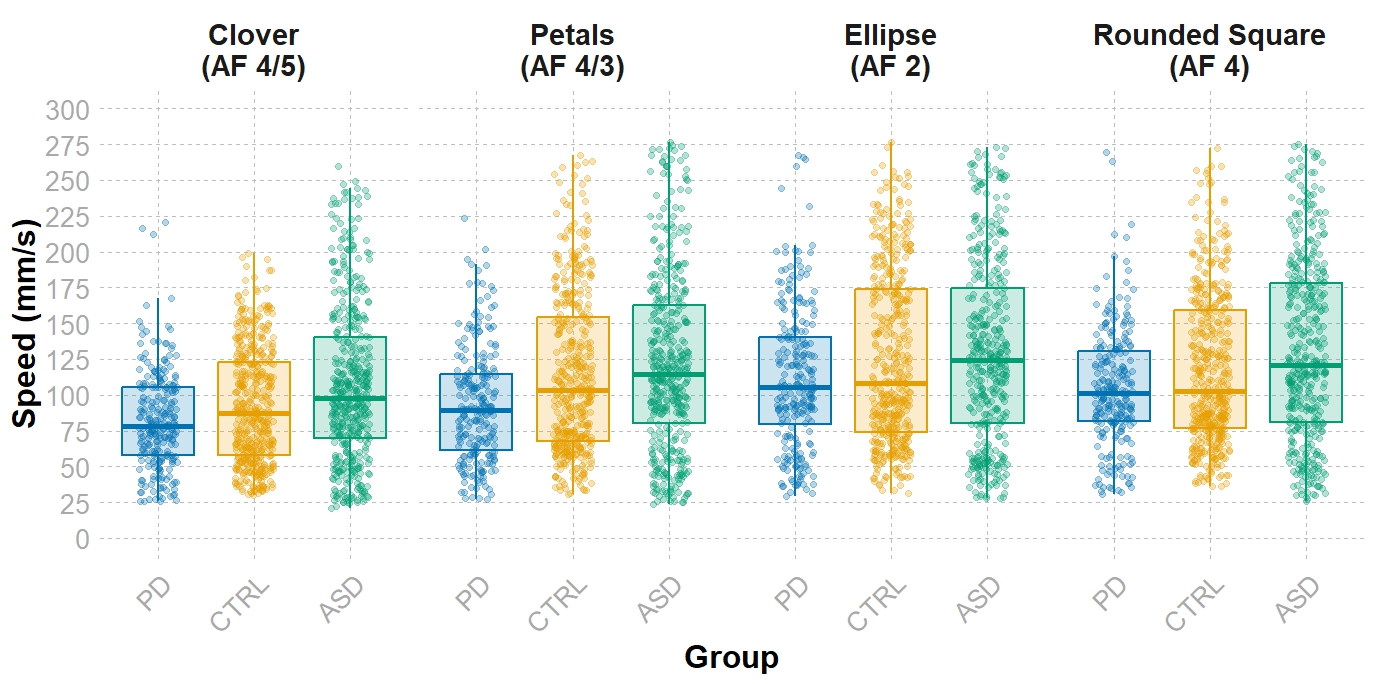


*Note.* Raw speed values are shown, after outlier removal but prior to the log transform applied for analysis. One data point per trial is plotted, calculated as the first-order derivative of the positional non-null data. Boxplots depict the median and interquartile range (IQR), with whiskers at 1.5 × IQR. Speed values have been converted from pixels/s to mm/s. AF = Angular Frequency; ASD=Autism Spectrum Disorder (green); CTRL=Control (orange); PD=Parkinson’s Disease (blue).

*Figure A2. Acceleration values for all trials across groups and shapes*


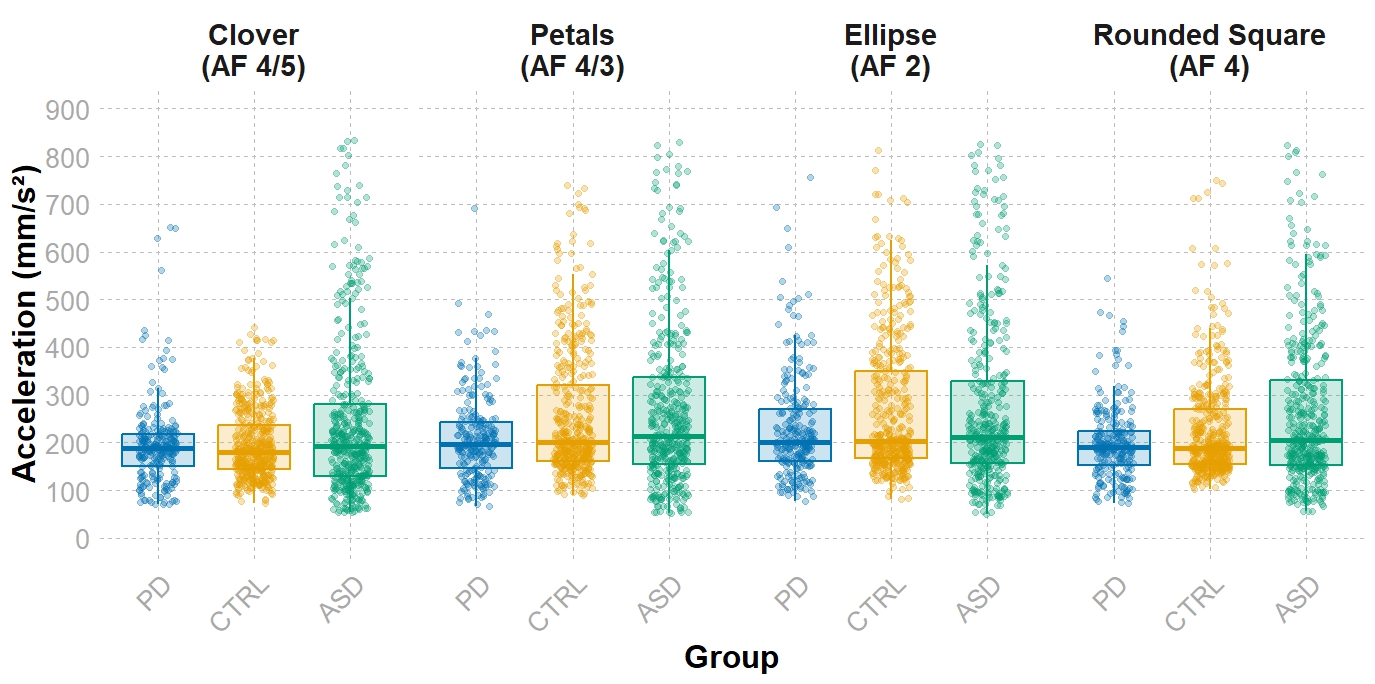


*Note.* Raw acceleration values are shown, after outlier removal but prior to the log transform applied for analysis. One data point per trial is plotted, calculated as the second-order derivative of the positional non-null data. Boxplots depict the median and interquartile range (IQR), with whiskers at 1.5 × IQR. Acceleration values have been converted from pixels/s^2^ to mm/s^2^. AF = Angular Frequency; ASD=Autism Spectrum Disorder (green); CTRL=Control (orange); PD=Parkinson’s Disease (blue).

*Figure A3. Jerk values for all trials across groups and shapes*


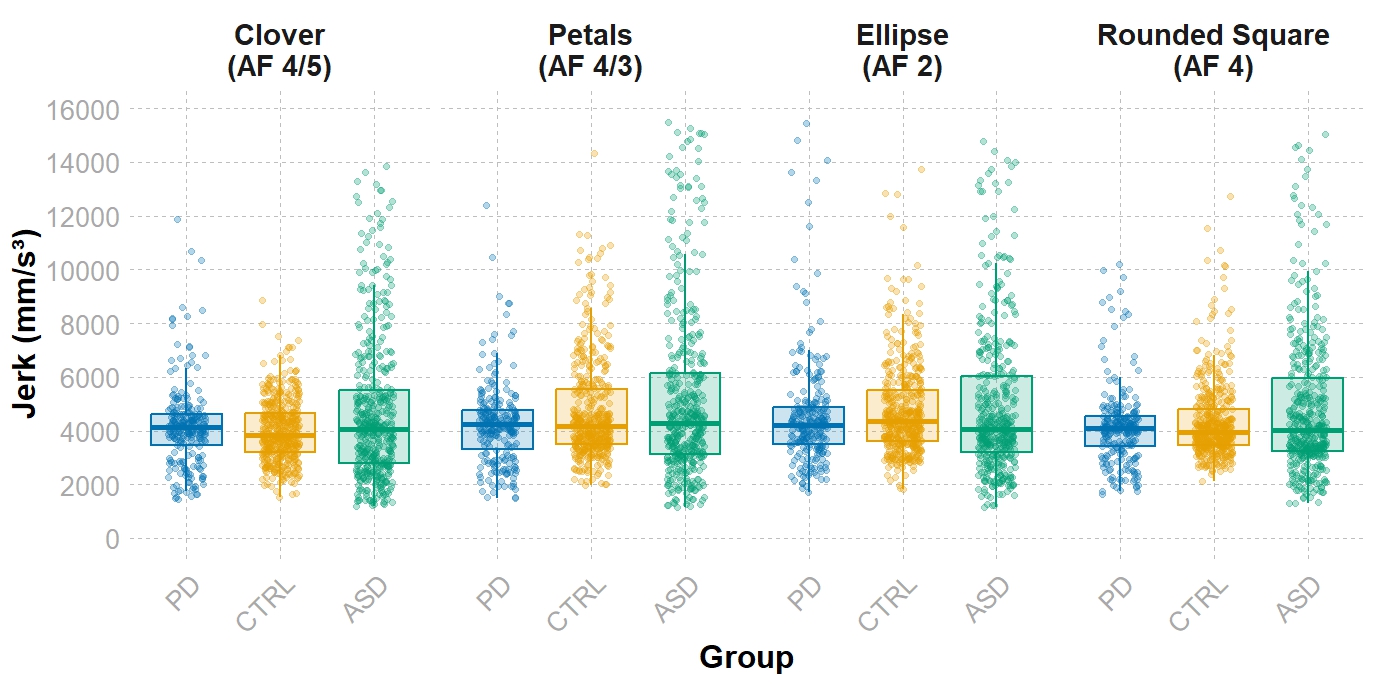


*Note.* Raw jerk values are shown, after outlier removal but prior to the log transform applied for analysis. One data point per trial is plotted, calculated as the third-order derivative of the positional non-null data. Boxplots depict the median and interquartile range (IQR), with whiskers at 1.5 × IQR. Jerk values have been converted from pixels/s^3^ to mm/s^3^. AF = Angular Frequency; ASD=Autism Spectrum Disorder (green); CTRL=Control (orange); PD=Parkinson’s Disease (blue).

*Figure A4. Sub-movements for all trials across groups and shapes*

*
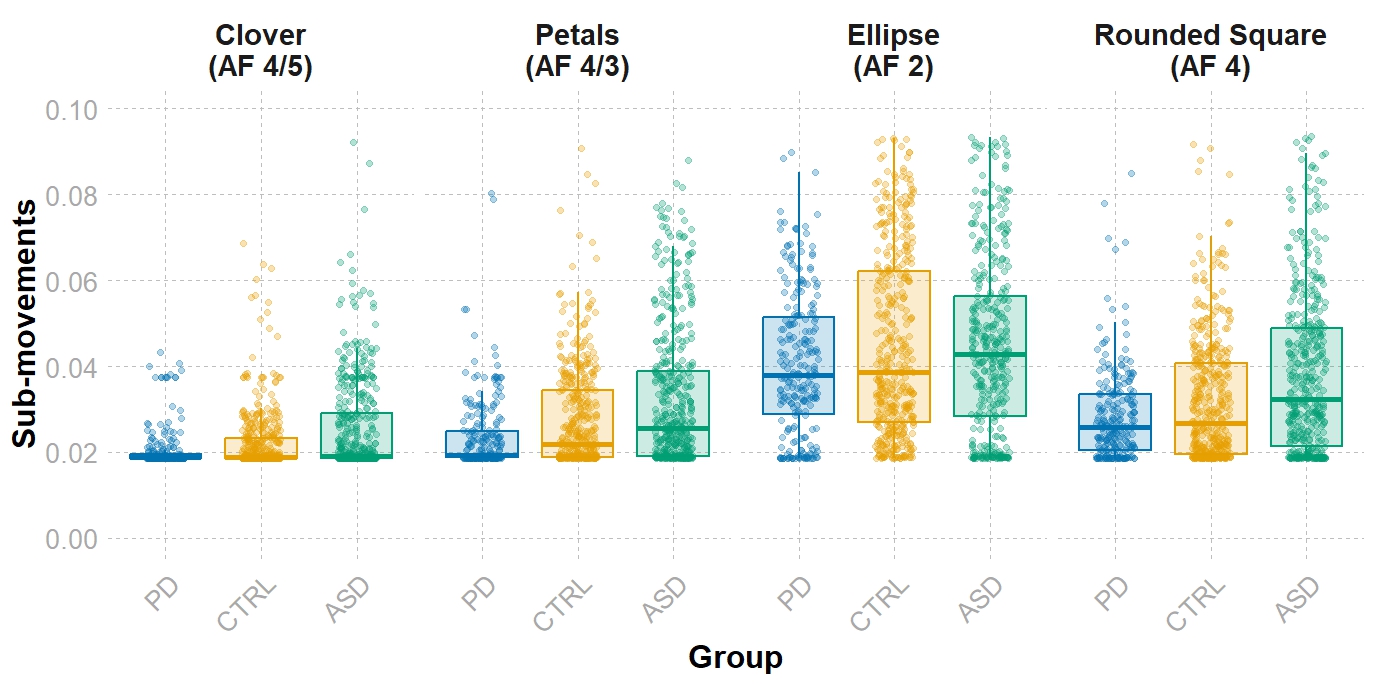
*

*Note.* Raw sub-movement values are shown, after outlier removal. One data point per trial is plotted, calculated as the percentage of time samples in which a change in acceleration sign was observed. Boxplots depict the median and interquartile range (IQR), with whiskers at 1.5 × IQR. A significant main effect was observed between ASD and PD, and ASD and CTRL. AF = Angular Frequency; ASD=Autism Spectrum Disorder (green); CTRL=Control (orange); PD=Parkinson’s Disease (blue).

*Figure A5. Speed modulation for all trials across groups and shapes*

*
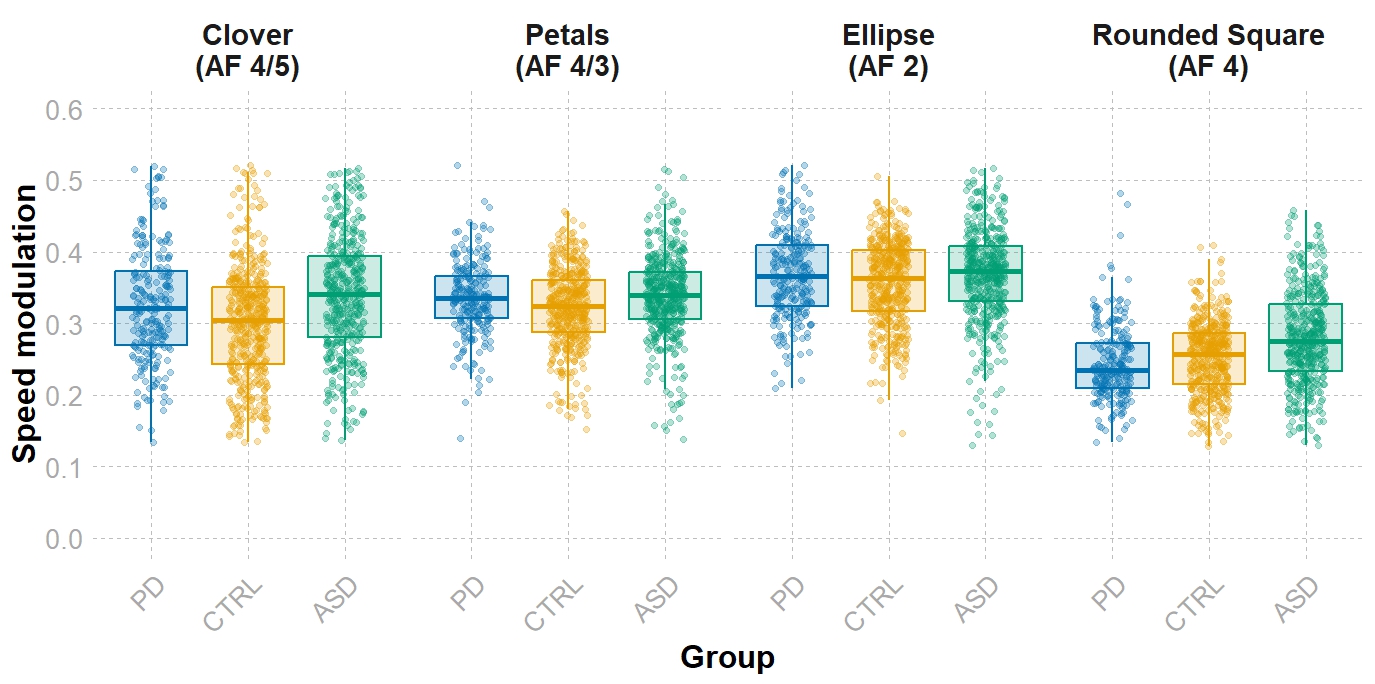
*

*Note.* Raw speed modulation values are shown, after outlier removal. One data point per trial is plotted, calculated as the gradient between tangential velocity and the current curvature of the shape being drawn, converted to an absolute value. Boxplots depict the median and interquartile range (IQR), with whiskers at 1.5 × IQR. A significant difference was found between ASD and CTRL, and ASD and PD, for the rounded square. AF = Angular Frequency; ASD=Autism Spectrum Disorder (green); CTRL=Control (orange); PD=Parkinson’s Disease (blue).

*Figure A6. Minimum speed values for all trials across groups and shapes*


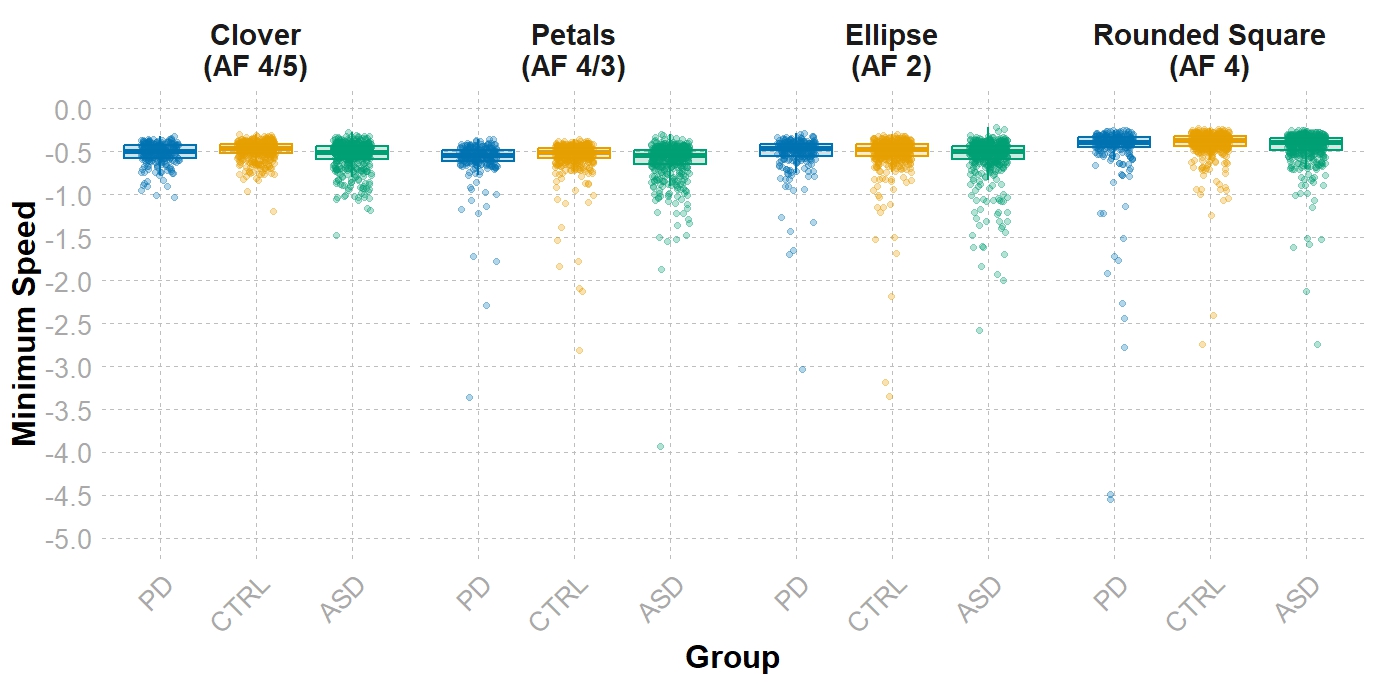


*Note.* Minimum speed values are shown, after outlier removal but prior to the reciprocal transform applied for analysis. This variable was calculated as the average of the bottom 10% of log velocity values produced in the speed modulation pre-processing stage, meaning they are represented on a logarithmic scale. One data point per trial is plotted. Each point represents one trial. Boxplots depict the median and interquartile range (IQR), with whiskers at 1.5 × IQR. AF = Angular Frequency; ASD=Autism Spectrum Disorder (green); CTRL=Control (orange); PD=Parkinson’s Disease (blue).

*Figure A7. Maximum speed values for all trials across groups and shapes*


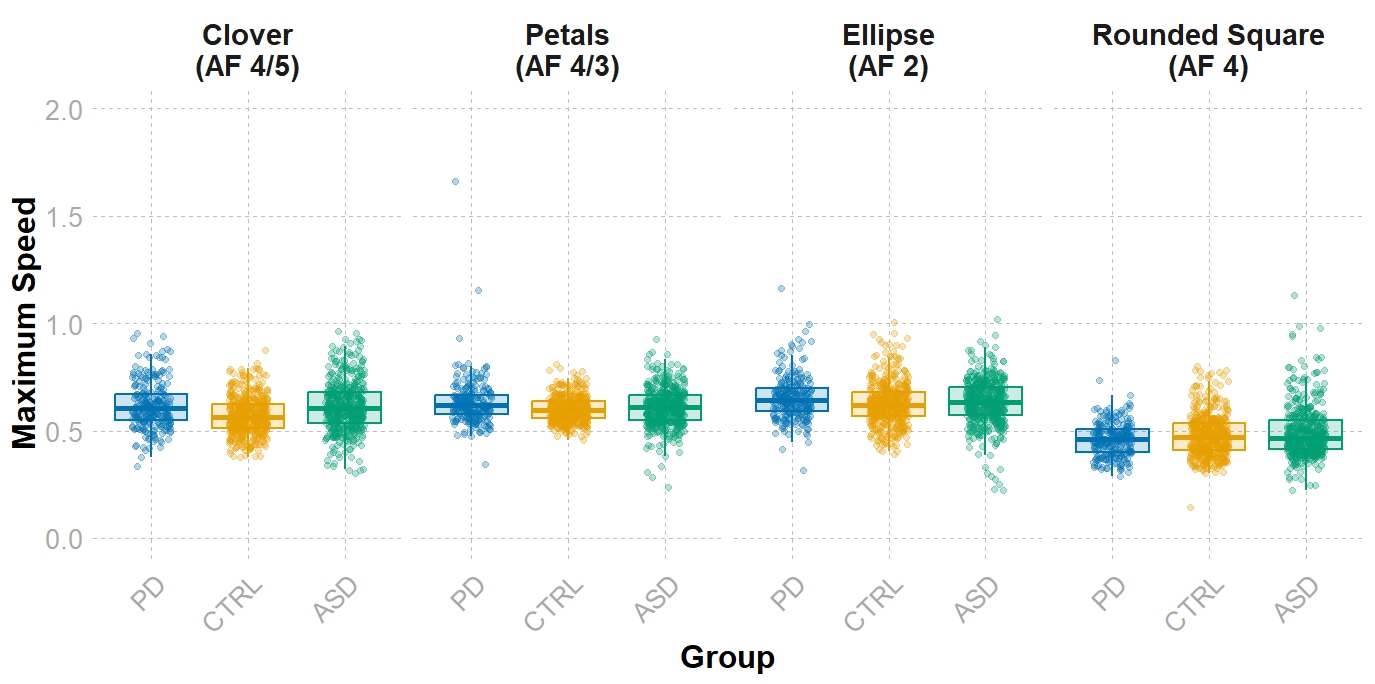


*Note.* Maximum speed values are shown, after outlier. This variable was calculated as the average of the top 10% of log velocity values produced in the speed modulation pre-processing stage, meaning they are represented on a logarithmic scale. One data point per trial is plotted. Each point represents one trial. Boxplots depict the median and interquartile range (IQR), with whiskers at 1.5 × IQR. AF = Angular Frequency; ASD=Autism Spectrum Disorder (green); CTRL=Control (orange); PD=Parkinson’s Disease (blue).

*Figure A8. SPARC values for all trials across groups and shapes*


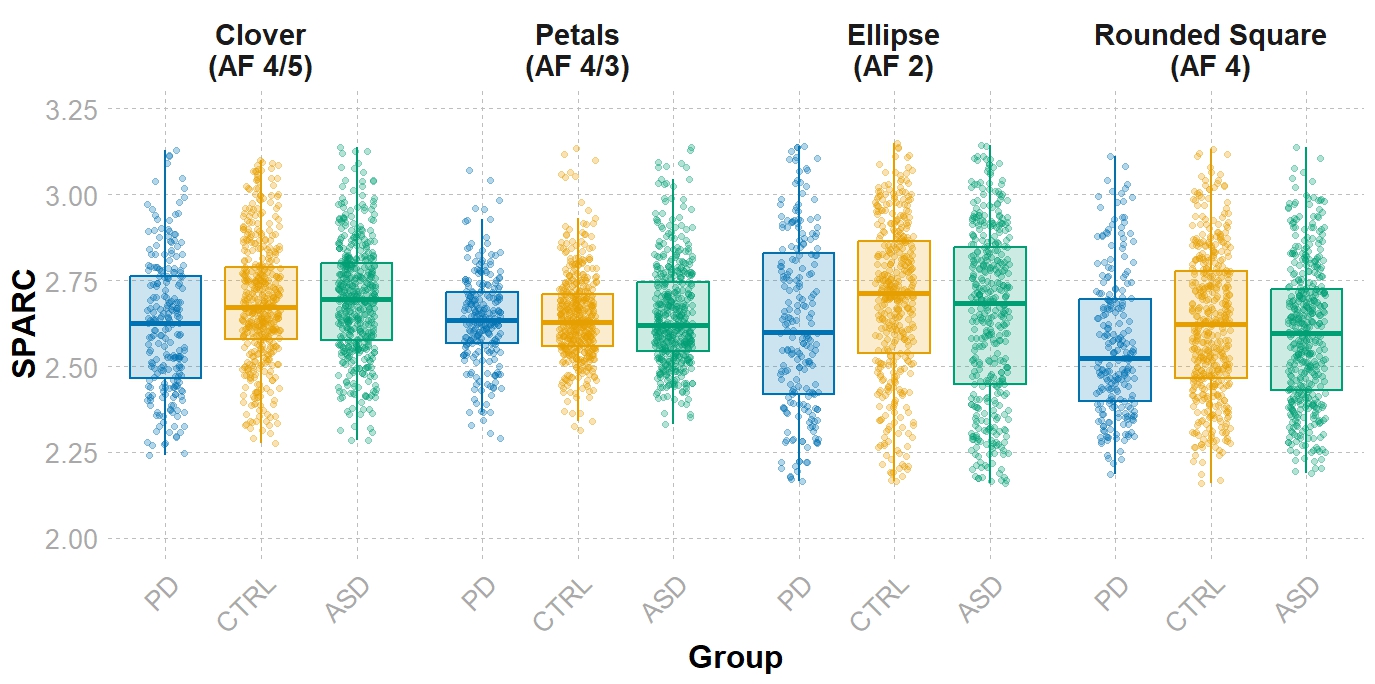


*Note.* Raw SPARC values are shown, after outlier removal. One data point per trial is plotted, calculated as the arc length of the magnitude spectrum arising from a Fourier transform of the speed profile. Boxplots depict the median and interquartile range (IQR), with whiskers at 1.5 × IQR. A significant difference was found between ASD and CTRL, and ASD and PD, on shape 4. AF = Angular Frequency; ASD=Autism Spectrum Disorder (green); CTRL=Control (orange); PD=Parkinson’s Disease (blue).

*Figure A8. Error measure values for all trials across groups and shapes*


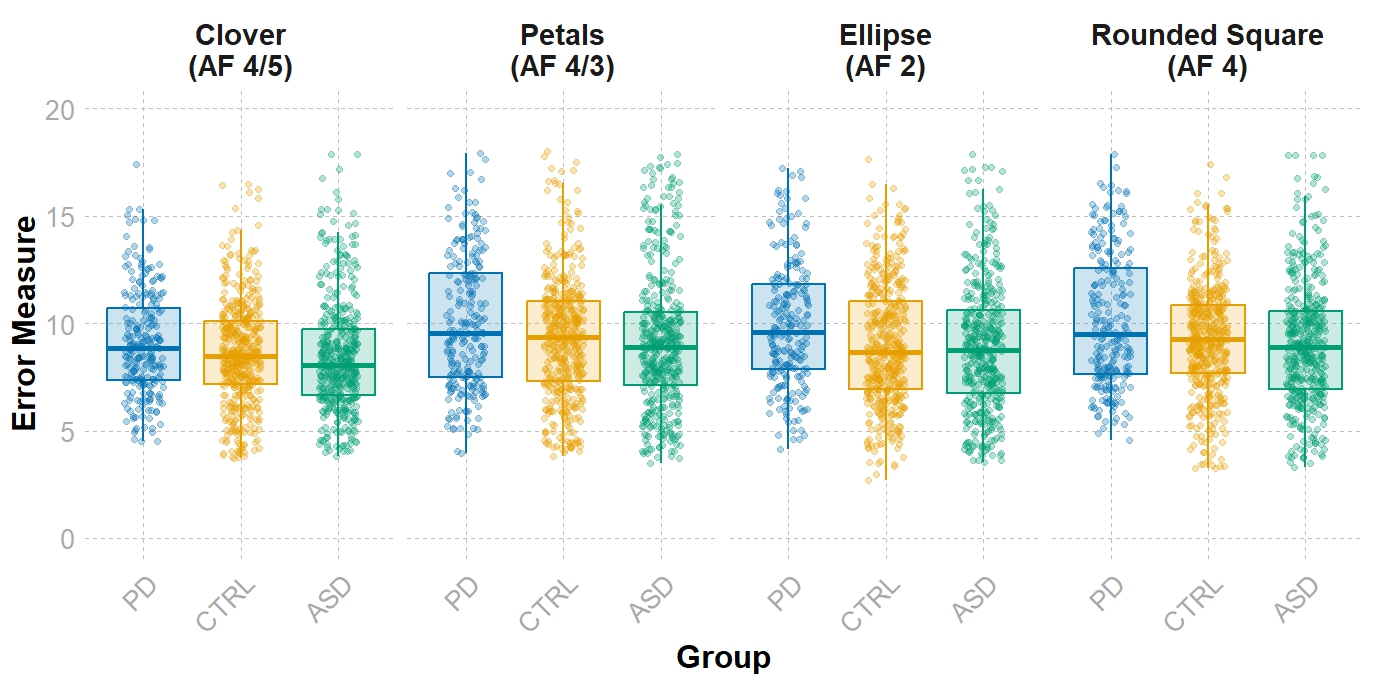


*Note.* Raw error measure values are shown, after outlier removal. One data point per trial is plotted, calculated as the absolute mean of the normal distance to the tangent of the nearest point on the shape’s curve. Boxplots depict the median and interquartile range (IQR), with whiskers at 1.5 × IQR. A significant difference was found between ASD and CTRL, and ASD and PD, on shape 4. AF = Angular Frequency; ASD=Autism Spectrum Disorder (green); CTRL=Control (orange); PD=Parkinson’s Disease (blue).
